# The Biological Performance of a Novel Electrokinetic-Assisted Membrane Photobioreactor (EK-MPBR) for Wastewater Treatment

**DOI:** 10.3390/membranes12060587

**Published:** 2022-05-31

**Authors:** Maryam Amini, Eltayeb Mohamedelhassan, Baoqiang Liao

**Affiliations:** 1 Department of Biotechnology, Lakehead University, 955 Oliver Road, Thunder Bay, ON P7B 5E1, Canada; 2 Department of Civil Engineering, Lakehead University, 955 Oliver Road, Thunder Bay, ON P7B 5E1, Canada; emohamed@lakeheadu.ca; 3 Department of Chemical Engineering, Lakehead University, 955 Oliver Road, Thunder Bay, ON P7B 5E1, Canada; bliao@lakeheadu.ca

**Keywords:** electrokinetic-assisted membrane photobioreactor, nutrient removal, wastewater treatment, phycoremediation

## Abstract

Developing an effective phycoremediation system, especially by utilizing microalgae, could provide a valuable approach in wastewater treatment for simultaneous nutrient removal and biomass generation, which would help control environmental pollution. This research aims to study the impact of low-voltage direct current (DC) application on *Chlorella vulgaris* properties and the removal efficiency of nutrients (N and P) in a novel electrokinetic-assisted membrane photobioreactor (EK-MPBR) in treating synthetic municipal wastewater. Two membrane photobioreactors ran in parallel for 49 days with and without an applied electric field (current density: 0.261 A/m^2^). Mixed liquid suspended soils (MLSS) concentration, chemical oxygen demand (COD), floc morphology, total phosphorus (TP), and total nitrogen (TN) removals were measured during the experiments. The results showed that EK-MPBR achieved biomass production comparable to the control MPBR. In EK-MPBR, an over 97% reduction in phosphate concentration was achieved compared to 41% removal in the control MPBR. The control MPBR outperformed the nitrogen removal of EK-MPBR (68% compared to 43% removal). Induced DC electric field led to lower pH, lower zeta potential, and smaller particle sizes in the EK-MPBR as compared with MPBR. The results of this novel study investigating the incorporation of *Chlorella vulgar* is in an electrokinetic-assisted membrane photobioreactor indicate that this is a promising technology for wastewater treatment.

## 1. Introduction

Wastewater treatment is a growing concern because wastewater contains pollutants such as nitrogen and phosphorus, which in excess can threaten wildlife and marine life [1,2]. Algae can assimilate these nutrients from wastewater and prevent eutrophication [3,4,5,6]. Phycoremediation, or biological treatment that utilizes algae for nutrient removal from wastewater, is one of the recent technologies gaining attention due to its low cost and environmental footprint [5,7,8]. Membrane photobioreactor technology (MPBR), as one of the biological wastewater treatment systems, is widely used for simultaneous wastewater treatment and microalgae production [9]. This technology has gained momentum due to promising nutrient removal and the high quality of effluent, together with the production of concentrated microalgae [10,11]. The biomass production of MPBRs has industrial applications, including biofuel, foods, and feeds [12,13,14].

Several studies on MPBR have been conducted by various research groups with different microalgae species and MPBR configurations [15,16,17,18]. These studies have demonstrated the advantages of MPBR systems for nutrient removal and microalgae biomass production compared to the conventional microalgae system [19].

*Chlorella vulgaris* (*C. vulgaris*) is an extensively used microalgae in MPBRs for wastewater treatment [8,20,21]. It is also produced for human nutrition and biodiesel feedstock applications [12,14,22]. *C. vulgaris* can grow in diverse environments [23,24,25] such as high temperature, e.g., up to 40 °C [14], acidic and alkaline (pH from 3 to 11.5) [20,21,22], light intensity [22], and high salinity. The most studied condition affecting their growth is light, but few studies focus on the effect of a low-voltage continuous electric field on photosynthesis and growth efficiency [14].

There are several parameters that affect the phycoremediation process [8]. Wastewater characteristics are one of the factors that have been studied. One recent study investigated the implications of urban wastewater concentration and induced stress on the growth of *Chlorella fusca* [26]. Using real wastewater instead of synthetic wastewater is another recent research focus on the growth study of *C. vulgaris* and its bioremediation of primary (PE) and secondary (SE) urban effluents [27]. Some reported researchers have focused on operating conditions such as hydraulic retention time (HRT), solid retention time (SRT), and turbulent pulsation [28,29,30]. 

Some studies investigated the effect of a low-voltage electric field on the growth and nitrogen and phosphorus removal efficiency of *C. vulgaris* [31,32]. Other research groups have studied the application of moderate and short-term electric fields in stimulating the growth and metabolism of *C. vulgaris* [13,33]. They found that in batch culture, a short-term applied electric field could improve the biomass growth of microalgae [13,33]. A study using an applied pulse electric field recently revealed the potential for increased lipid content from *C. vulgaris* [34].

Studies that incorporate electric fields in membrane bioreactors (MBR) have found improved chemical oxygen demand removal (COD) as well as nitrogen and phosphorus removal were observed by applying a short-term electric field in membrane bioreactors (MBR), where activated sludge was used [35,36,37,38]. To date, only one study has integrated an electric field into an MBR with algae and activated sludge [2]. However, the combination of an electric field with a membrane photobioreactor utilizing algae as biomass has not yet been investigated.

The current study examines the effect of a low-voltage continuous electric field on the microalgae growth rate, biomass quality, and overall nutrient removal (N and P) of an MPBR with *C. vulgaris* in treating synthetic municipal wastewater. An MPBR and an electrokinetic-assisted MPBR (EK-MPBR) with *C. vulgaris* were operated in parallel for 49 days to investigate the electric field effects on biomass production, biomass productivity, COD removal, and nutrient (N and P) removals. This is the first study on EK-MPBR, and the results demonstrate that it is a promising technology that simultaneously removes nutrients and reproduces microalgae.

## 2. Materials and Methods

### 2.1. Chemicals 

All chemicals were purchased from Sigma-Aldrich (Merck, Darmstadt, Germany). Deionized water was prepared in the laboratory.

### 2.2. Microalgae and Culture Conditions

The microalgae *C. vulgaris* was purchased from the Canadian Phycological Culture Centre of the University of Waterloo, ON, Canada. The medium solution was prepared with the following composition and amounts in 1 L of solution [39,40]:

0.66 g NH_4_Cl, 0.625 g MgSO_4_, 0.1105 g CaCl_2_·2H_2_O, 0.1142 g H_3_BO_3_, 0.0498 g FeSO_4_·7H_2_O, 0.0882 ZnSO_4_·7H_2_O, 0.0144 g MnCl_2_·4H_2_O, 0.0118 g Na_2_MoO_4_·2H_2_O, 0.0157 g CuSO_4_·5H_2_O, 0.004 g CoCl_2_·6H_2_O, 0.64 g ethylenediamine tetraacetic acid (EDTA)-2 Na·2H_2_O, 0.6247 g KH_2_PO_4_, 1.3251 g K_2_HPO_4_. All components were added to deionized (DI) water and insulated to avoid contamination. The cultivation continued for 25 days to reach the desired concentration of 1.2 g/L of dried biomass under continuous aeration and light illumination at room temperature.

### 2.3. Operating Conditions

The feed of the reactors was a synthetic municipal wastewater effluent (after biological chemical oxygen demand (COD) removal). The amount of the trace element in one liter feed summarized in Table 1 included 0.0025 g NaCl, 0.082 g MgSO_4_·7H_2_O, 0.005 g CaCl_2_·2H_2_O, 0.02490 g FeSO_4_·7H_2_O, 0.00044 ZnSO_4_·7H_2_O, 0.00022 g MnCl_2_·4H_2_O, 0.00126 g Na_2_MoO_4_·2H_2_O, 0.00039 g CuSO_4_·5H_2_O, 0.00041 g CoCl_2_·6H_2_O, 0.09553 NH_4_Cl, 0.01537 g KH_2_PO_4_, 0.3 g NaHCO_3_ and 0.01874 g Glucose. All the components were added to distilled water. The reactors were fed semi-continuously with a liquid level controller sensor and a peristatic pump, and the feed was kept in a refrigerator at 4–5 °C. The level of the suspensions in the reactors was kept constant by a level sensor (LC40, Flowline Inc., Los Alamitos, CA, USA), and feed pumped by a peristaltic pump (Model 77122-12, Masterflex^®^C/L^®^PWR, Cole-Parmer, Vernon Hills, IL, USA). The permeation pumps were programmed to operate in 3-min-on and 2-min-off modes for permeation and relaxation to control membrane fouling. The reactors were operated for 49 days, and hydraulic retention time (HRT) and solid retention time (SRT) were maintained at 2.5 days and 30 days, respectively. During the operation, HRT was controlled by applied membrane flux. Once the transmembrane pressure (TMP) of the MPBRs reached about 30 kPa, physical cleaning was used to remove the foulant layer of the membrane module. The pH of the feed for the MPBR was adjusted using NaOH and HCl solutions. 

### 2.4. Experimental Set-Up

**Figure 1:**
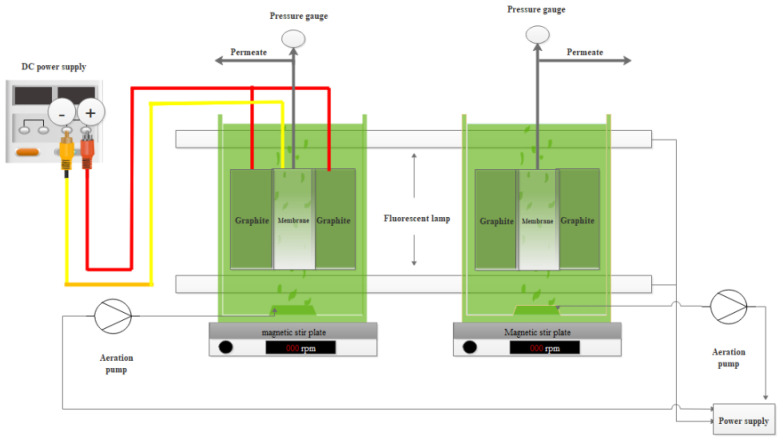
Schematic of lab-scale MPBR and EK-MPBR set-up.

The experiments were conducted using two lab-scale submerged MPBRs. The reactors were filled with 10 L of culture medium. White LED lamps were used to provide illumination. The reactors were put in a magnet stir to mix the algal solution slightly. The schematic diagram of the experimental set-up is shown in Figure 1. Table 2 summarizes the basic parameters of the membrane module and the operating conditions. Two flat sheet rectangular graphite electrodes and two stainless steel meshes were placed in the reactors. The EK-MPBR is connected to a DC power supply system, and the MPBR has the same configuration but without DC power supply. The graphite and stainless steel sheets were connected to positive and negative poles of a DC power supply (B & K precision’s, Taiwan), respectively. A constant DC electric field was applied to the microalgae for the entire operation for the EK-MPBR.

### 2.5. Zeta Potential and Routine Analysis

The zeta potential of the flocs was determined by using a NanoBrook ZetaPlus (Brookhaven, NY, USA). The samples were diluted in 1 mM KCl solution. Each sample was tested at least twice to confirm the zeta potential value. Smoluchowski’s equation was used to determine the zeta potential [41]. A dissolved oxygen (DO) meter (Model 407510, Extech, Nashua, NH, USA), a pH meter (pH 700, Oakton, VA, USA), and a thermometer were used to measure the DO, pH, and temperature of the suspension in the reactor.

### 2.6. Determination of Biomass Characteristics

The MLSS and COD were measured using the standard method [42]. Biomass productivity was calculated based on the following equation [43]:

(1) $$r_{x}=X\times \frac{Q_{\mathrm{waste}}}{V_{\mathrm{MPBR}}}=\frac{X}{\mathrm{SRT}}$$

where, r_x_ is biomass productivity (mg/L·d); X is the average biomass concentration (g/L), which in this study equals to average MLSS; Q waste is the reactor biomass wasting rate (L/d), and VMPBR is its working volume (L).

Total nitrogen and phosphorus (TN, TP) were monitored on samples taken every other day. Each sample was duplicated, and the values reported are the averages for each sample. Both TN and TP were measured by spectrophotometry using the alkaline potassium persulfate digestion-UV spectroscopy method [39,44] and the ammonium molybdate spectroscopy method, respectively [44].

### 2.7. Particle Size Distribution and Microalgae Structure

The particle size distribution was measured by a Malvern Mastersize 2000 instrument (Worcester, UK) with a detention of 0.02–2000 µm. Each sample was measured in triplicate. The range of laser obscuration was 0.1–0.4%.

The structure of the algal cell was studied by an inverted microscope (Olympus IX51, Tokyo, Japan). For each picture, the samples were dropped onto a slide, followed by dispersion with a cover slide. To gather images, each sample was randomly photographed with a digital camera connected to a microscope.

### 2.8. Statistical Analysis

The statistical difference of the parameters in MPBR and EF-MPBR was determined by two-sample *t*-tests, with the alpha significance level at 0.05 (*p* = 0.05).

## 3. Results and Discussion

In this study, the performance of the electrokinetic-assisted photobioreactor on phycoremediation is investigated and compared with a control photobioreactor. The effect of the electric field is classified in terms of biomass production, nutrient removal, zeta potential, pH, and floc morphology.

### 3.1. Effect of EF Treatment on Biomass Production

**Figure 2:**
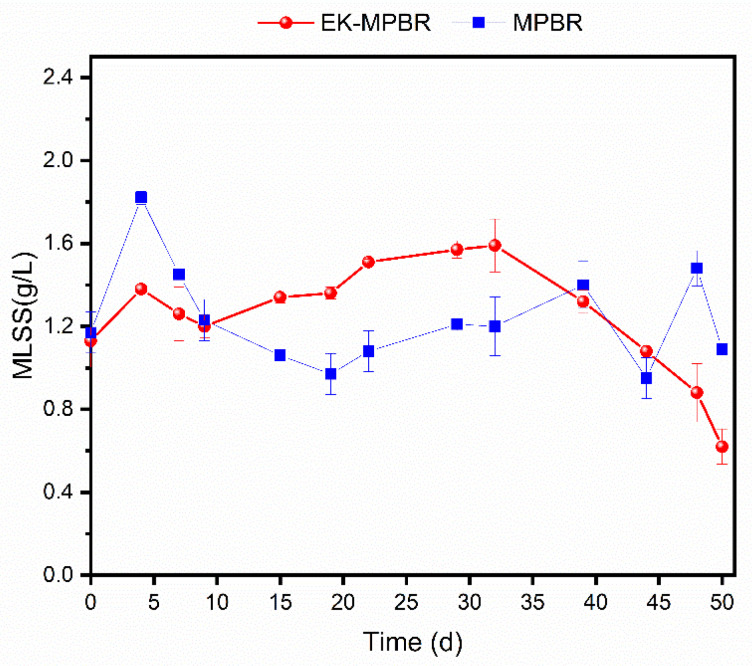
Variation of biomass production for MPBR with and without applied electric field over the experimental period.

We investigated the effect of the applied electric field on algae growth in photobioreactors. The MLSS concentration was measured and used as an indicator of algal growth throughout the study. The time course measuring the MLSS concentration of the reactor is shown in Figure 2. Both MPBRs operated in parallel with an initial concentration of 1.16 ± 0.4 g/L of MLSS. The biomass value and productivity of the control reactor (MPBR) varied from 0.97 g/L to 2.12 g/L and 32.33–70.66 g/Ld, respectively. For the EK-MPBR, the corresponding values were lower than the control, with biomass ranging from 0.62 g/L to 1.59 g/L of MLSS and productivity ranging from 18–53 mg/Ld. However, these values were not significantly different (*p* > 0.05).

In the EK-MPBR, MLSS fluctuation over the first 39 days of the experiment was smoother and relatively higher compared to the control MPBR. This improved productivity suggests that the applied electric field stimulated the growth of microalgae. The present study is consistent with the findings of others that the electric field increased the productivity of *Chlorella vulgaris* by enhancing the transport of substances across the algae cell membrane [45,46]. The hormetic response of low-dose stimulation and high-dose inhibition seen in Figure 2 is an adaptive cell response that is stimulatory in the short term and inhibitory in long-term exposure [47] was also observed in the pre-treatment of *Chlorella vulgaris* with the application of a short-term moderate electric field [13]. 

Corpuz et al. studied the effect of a long-term applied electric field bioreactor, where they observed a similar trend in the Algae-Activated sludge bioreactor [2]. In the study of Corpuz et al., it was mentioned that more prolonged exposure to the electric field inhibited microalgae growth starting on day 28. This retardation of microalgae, which is caused mainly by electrochemical reactions around the cathode, was delayed in this study until day 39. Indirect oxidation due to the modified design of the cathode in our study could play a role in growth inhibition caused by hydroxyl radicals. By placing the cathode behind the membrane, the released ions in the permeate could be removed by the permeate pump, and therefore, their accumulation over time is controlled. The indirect oxidation effect on the molecular level in MPBRs could be the focus of future studies. 

### 3.2. Nutrient Removal and Wastewater Treatment Potential

**Figure 3:**
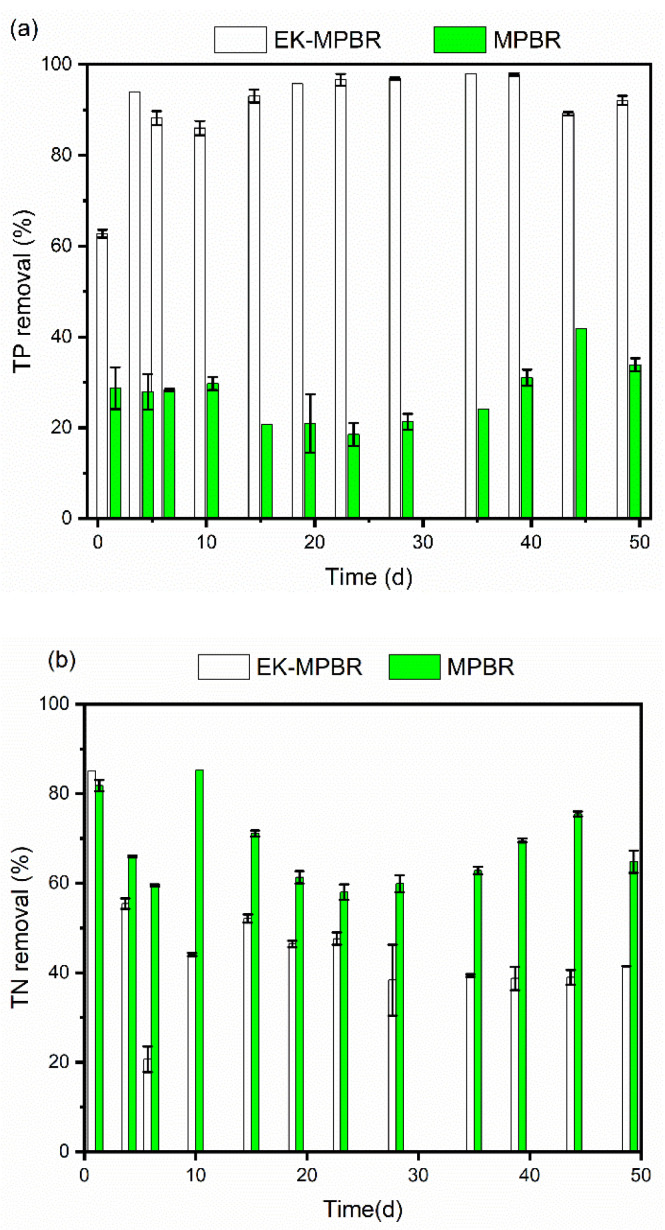

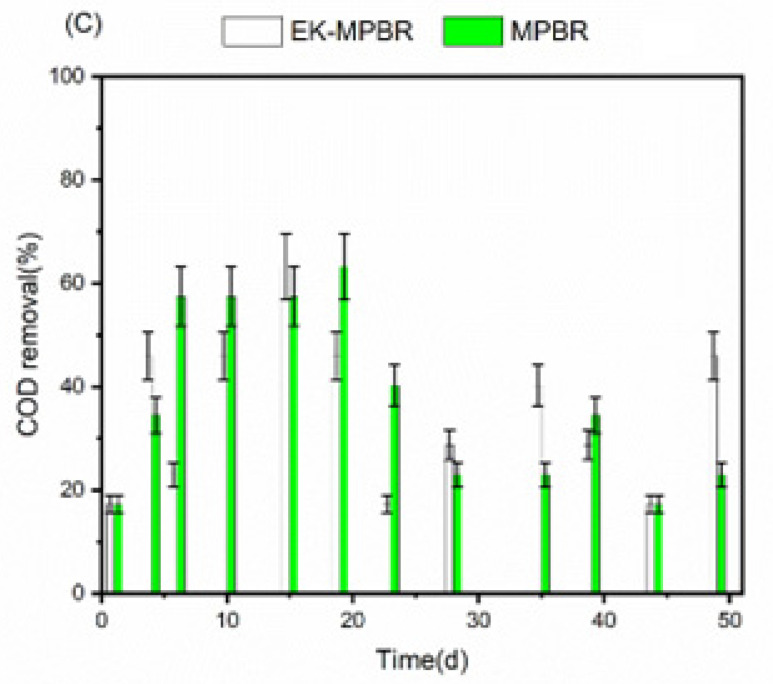
Nutrient removal of MPBR and EK-MPBR: (**a**) comparison of TP removal efficiency, (**b**) TN removal efficiency, and (**c**) COD removal efficiency.

The wastewater treatment performance of EK-MPBR was compared to the control MPBR to determine electric field efficiency in terms of nutrient removal from the wastewater. The efficiency of a phycoremediation system is defined by how well algae can remove nitrogen, phosphorus, and COD from wastewater. Figure 3 shows the percentage of nutrient removal of EK-MPBR and MPBR over time. The concentrations of N, P, and COD in the influent were maintained constant at the levels of 25n ± 2 mg/L, 3.5 ± 0.3 mg/L, and 20 ± 2.5 mg/L, respectively. EK-MPBR showed a statistically significant higher phosphorous removal with an overall removal of 97.98n ± 0.02% (*p* < 0.05). This demonstrates the advantage of EK-MPBR for phosphorus removal compared to the overall removal of 41.81 ± 0.05% of the control reactor. The main two phosphorus removal mechanisms in algal systems are biomass adsorption and precipitation of phosphorus [2,25]. In an electric field-assisted system, electrochemical oxidation on the surface of the electrodes and electrochemical reactions in the suspension can also contribute to phosphorus removal. Given a pH range of 7.5 to 8.5 for EF-MPBR, phosphorus adsorption on the surface of the anode (graphite) is not the dominant mechanism [48]. The improved phosphorus removal in EK-MPBR can be attributed to the occurrence of electrochemical reactions in the suspension and the overall ion strength in the mixed liquor solution [33,49]. A recent study showed that in biomass combined with activated sludge, the applied electric field improved phosphorus removal by 65% compared to its control reactor and was mainly due to electrochemical reactions [2]. The released aluminum ions from the aluminum anode and the generation of phosphate aluminum complex contribute to the removal of phosphorus [50,51]. Although a number of studies have evaluated the effect of electric field on MBRs [2,33,52,53], its effect on molecular adsorption in MPBRs needs to be verified in future studies. 

Limited phosphate concentration in the suspension in EK-MPBR can lower the biomass productivity and removal efficiency caused by a low biomass concentration [54]. However, this is in contrast to the MLSS concentration (Figure 2). As such, it is likely that the stimulating effect of the electric field outweighed the inhibitory effect of P depletion. Furthermore, the limited phosphate concentration in the suspension in EK-MPBR is beneficial for reducing membrane fouling. The correlation between P depletion and biofilm growth has been reported by other studies [55,56]. The low concentration of P, ranging from 0.05 mg/L to 1.09 mg/L in EK-MPBR, is below the concentration needed for biofilm growth compared to the control MPBR, ranging from 1.79 to 2.39 mg/L [49]. This suggests the existing potential of EK-MPBR for enhanced membrane performance. 

**Figure 4:**
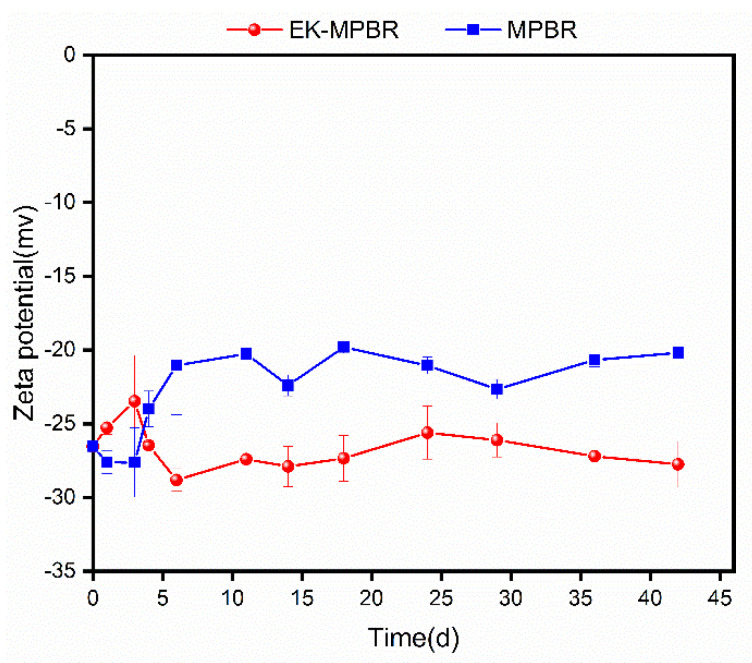
Zeta potential variation during EK-MPBR and MPBR operations.

Despite the significant phosphorus removal of EK-MPBR, the nitrogen removal efficiency of EK-MPBR was depressed to some extent. The better phosphorous removal efficiency compared to the nitrogen removal efficiency in EK-MPBR might be because of their different removal mechanisms. At the water-oxide interface, phosphate removal utilizes an inter-sphere adsorption mechanism that is less affected by ionic strength as compared to nitrogen removal, which has an outer-sphere adsorption mechanism [48]. As shown in Figure 3b, EK-MPBR TN removal ranged from 17.82% to 85%, whereas in control MPBR, TN was removed by 58% to 85% (*p* < 0.05). The concentration of TN in the influent was kept at a constant value of 25 ± 2 mg/L for both reactors. The lower nitrogen removal efficiency agrees with other studies that showed that the electric field might interfere with the nitrogen removal process [33,57,58]. The fluctuation over the 49 days in TN removal in EK-MPBR could be attributed to the change of MLSS concentration (Figure 3a), zeta potential (Figure 4), and ionic strength of the suspension, which may have interfered with nitrification. The lower nitrogen removal in EK-MPBR due to the presence of an electric field agrees with the study by Zhang et al. [33]. The potential changes in ionic properties of the microalgae could be due to the electrochemical reactions and the incorporation of the electric field, which further have an inhibitory impact on the removal of total nitrogen [33]. However, both EK-MPBR and MPBR showed nitrogen removal comparable to other studies (Table 3).

Figure 3c represents the COD concentration over the experimental period. The influent COD concentration for both reactors was kept constant at 20 ± 1.8 mg/L. The COD reduction can be attributed to the thickness of the biofilm, electrochemical oxidation of organic substances, and oxidation of the organic compounds by electrochemically generated oxidants such as hydrogen peroxide [2]. In this study, due to the induced multiple factors, further investigation is needed before highlighting any underlying reasons as the main mechanism of COD removal in EK-MPBR when comparing it with MPBR.

### 3.3. Effect of EF on the Physiology of Microalgae

Zeta potential, pH, and the morphology of biomass are measured as indicators of changes in physiology under the electric field and their effect on the phycoremedation efficiency [60,61]. In both MPBRs, the zeta potential remained negative over the experimental period. Zeta potential is dependent on factors such as pH and ion type and strength [62]. In alkaline conditions, zeta potential increases (i.e., becomes more negative) with pH increase as particles are surrounded by more negative charge in the suspension. The zeta potential values, represented in Figure 3, along with the pH data depicted in Figure 4, agree with the above statement.

The zeta potential in the control MPBR was higher than that of EK-MPBR. This can be attributed to the higher pH in the reactor compared to EK-MPBR [62]. Furthermore, zeta potential is a function of other factors, such as the composition and concentration of metabolites in the suspension [62]. This could explain the fluctuating behavior, especially over the first days of the operation, when the microalgae have unstable conditions due to adaptation to the new environment.

Surface charge, as represented by the zeta potential here, can contribute to the nutrient uptake efficiency of the system [63]. In alkaline solution, the predominant phosphate ions are ${\mathrm{HPO}}_{4}^{2-}$ and ${\mathrm{PO}}_{4}^{3-}$ [64]. The higher the surface charge, the stronger the electrostatic repulsion would interfere with the adsorption of these ions on the algal cell surface [64,65]. Reportedly, the lower surface charge positively affects the adsorption of the orthophosphate by *C. vulgaris* [65].

Sedimentation is another mechanism of phosphorus removal from wastewater that is also affected by the surface charge and electrostatic repulsion [66,67]. The phosphate ions form complex salts in wastewater, such as calcium phosphate in the form of sediments [67]. The lower surface charge would enhance sedimentation and phosphorus removal. Therefore, as shown in Figure 4, the decreased zeta potential in EK-MPBR (−27 compared to −20 mv in MPBR) can be attributed to the better phosphorus removal efficiency in EK-MPBR (97.98 ± 0.02% in EK-MPBR compared to 41.81 ± 0.05% in MPBR).

The nitrogen removal, however, was affected differently by the surface charge. Due to the increased surface charge, the adsorption of negatively charged hydroxyl ions (OH) that are part of the denitrification process could be decreased [68]. The effect of the electric field on this removal pathway can be further verified through electrochemical analysis of the cells in future studies.

**Figure 5:**
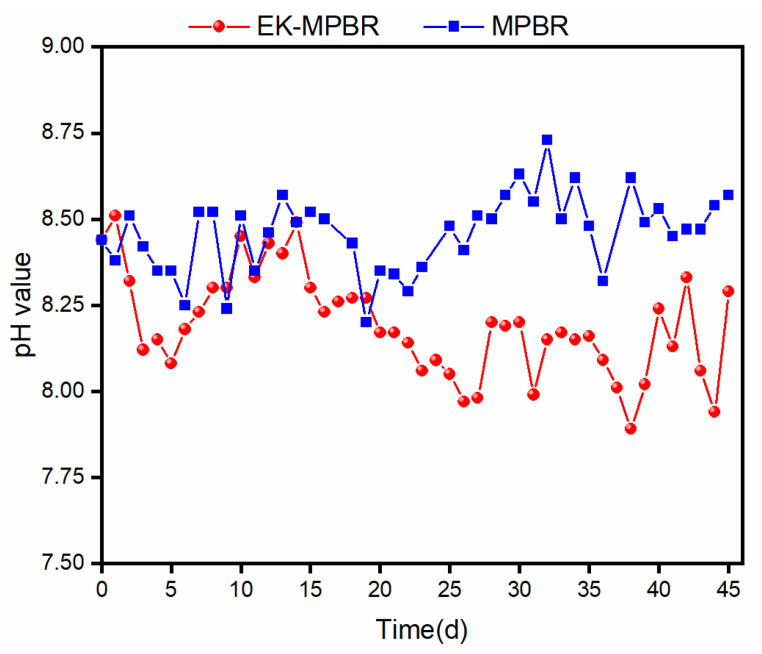
pH variation during EK-MPBR and MPBR operations.

The variation of pH with time is shown in Figure 5. While both reactors started with the same pH, the applied electric field lowered pH over time. The electrochemical reaction around the cathode is a determining factor [69]. In alkaline conditions, the following reaction at the cathode can be expected:

(2) $$2H_{2}O+2e^{-}\;\to \;H_{2}\left(g\right)+2{\mathrm{OH}}^{-}\;\left(\mathrm{aq}\right)$$

As a result of this electrolysis reaction, the pH near the cathode is expected to increase under the applied electric field. However, the results show that other factors may be involved in pH changes. One of the underlying reasons for pH changes could be the materials used and impurities that arise during the strengthening of the carbon in the manufacturing process, which could be released from the electrode to the suspension when placed under the electric field [70].

**Figure 6:**
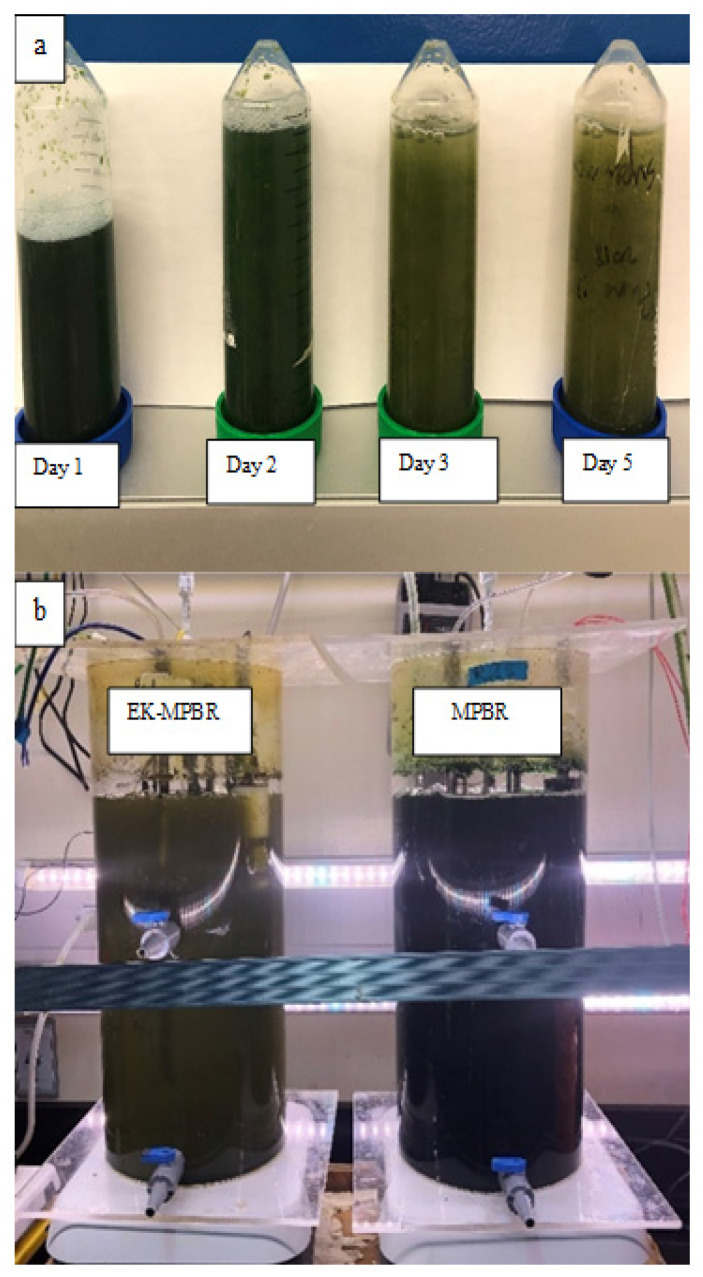
The color of microalgae grown in (**a**) EK-MPBR from day 1 to day 5 and (**b**) EK-MPBR and MPBR at day 20.

pH is also sensitive to the mechanism of microalgae growth. Considering glucose in the influent, the suspension provides a mixotrophic and/or heterotrophic culture for *C. vulgaris* growth. In both cases, mixotrophic and heterotrophic, pH depends on the microalgae’s preferred growth kinetics. While the pH remained unchanged in the mixotrophic condition, heterotrophic culture showed a gradual decrease in the suspension [71]. As shown in Figure 6, the lighter suspension over time under the electric field demonstrates heterotrophic dominancy, and therefore decreased pH.

**Figure 7:**
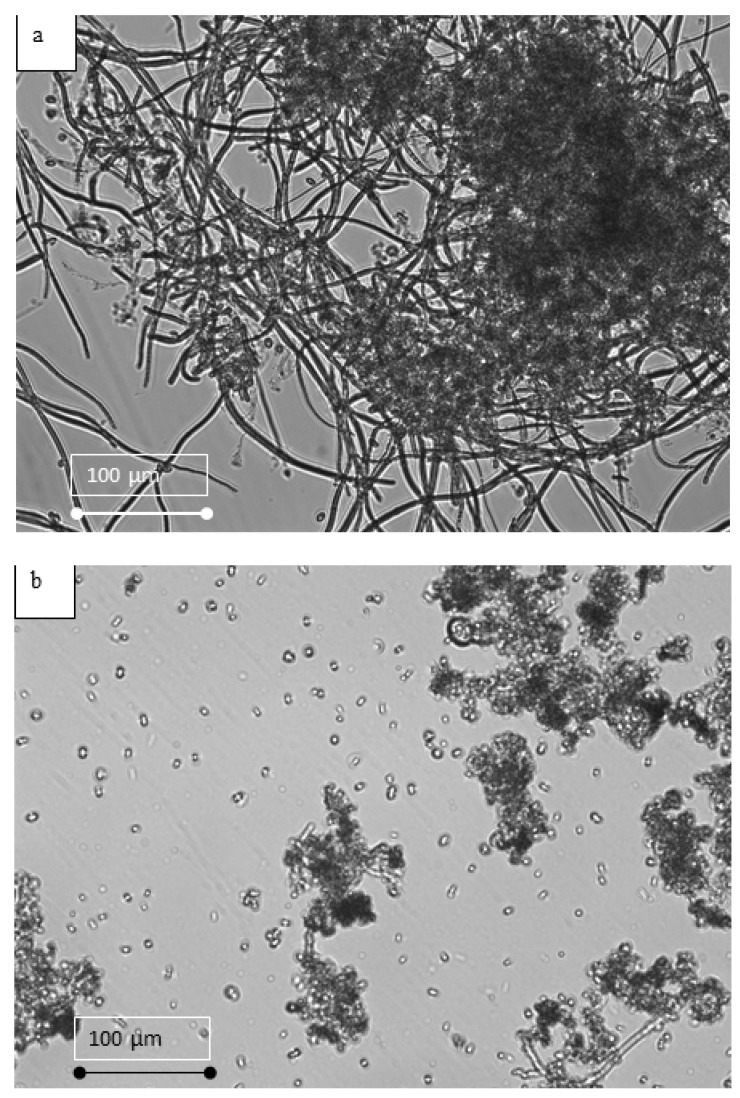
Microscopic images representing the morphology of *C. vulgaris* in (**a**) MPBR and (**b**) EK-MPBR.

**Figure 8:**
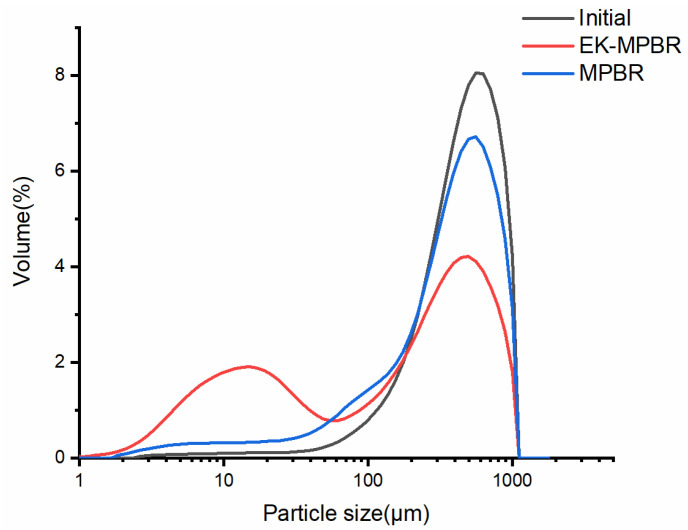
Particle size distribution of the floc suspension in the MPBR and EK-MPBR.

**Figure 9:**
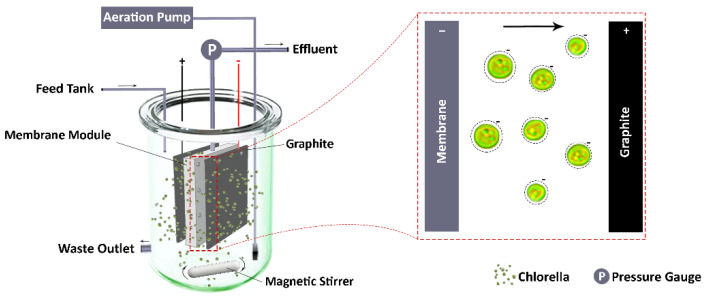
Conceptual figure of the electrophoresis movement of charged particles toward the opposite electrode in EK-MPBR.

Figure 7 shows the morphology of the suspensions in MPBR and EK-MPBR. One of the main objectives of this investigation is to study possible morphology changes due to the applied electric field. As shown in Figure 6, the floc size in the EK-MPBR is smaller than that of the MPBR. This agrees with the higher fraction of smaller particles seen in the particle size distribution (PSD) analysis (Figure 8). The fraction of the smaller particles in EK-MPBR (Figure 8) can be attributed to floc breakage and disintegration due to the applied electric field and the electrophoresis phenomenon. Electrophoresis and movement of the charged particles could result in more breakage of the flocs [49]. In electrophoresis, the charged particles tend to move toward the electrode with the opposite charge, which can cause collisions and smaller particle formation. This potentially explains the formation of smaller flocs in EK-MPBR. The conceptual image of the phenomena is presented in Figure 9.

## 4. Conclusions

A modified membrane photobioreactor that incorporated a low-voltage electric field and the algae *C. vulgaris* was developed. This novel study compared the biomass production and the nitrogen and phosphorus removal efficiency of *C. vulgaris* in the electrokinetic-assisted membrane bioreactor with that of the control.

The biomass production in the EK-MPBR was comparable to that in the MPBR. Nutrient removal was lower and significantly higher in EK-MPBR for total nitrogen and total phosphorus, respectively. This can be explained by electrochemical reactions around the electrodes. The results also showed that increased cell charge and formation of smaller particles under the applied electric field was observed, which may affect biomass production.

The work presented here has implications for future studies of the electric field in MPBRs and may help modify the design of membrane photobioreactors for membrane fouling control. Further research identifying the mortality rate of the algae under different applied currents molecular changes and electrokinetically affected assimilation efficiency of the cells will be beneficial for improving electro-phycoremediation techniques in wastewater treatments.

## Figures and Tables

**Table 1:** Characteristics of synthetic wastewater.

Water Quality Index	Average Value (mg/L)
Nitrogen	25 ± 2
Phosphorus (PO_4_ − P)	3.5 ± 0.3
Chemical Oxygen Demand	20 ± 2.5

**Table 2:** Specification of the membrane module and operating condition.

**Membrane Module**	**MPBR**	**EK-MPBR**
Total membrane surface area	0.03 m^2^	0.03 m^2^
Membrane materials	Polyvinylidene fluoride (PVDF)	Polyvinylidene fluoride (PVDF)
Membrane type	Flat sheet	Flat sheet
Mean membrane pore size	0.4 µm	0.4 µm
**Operational Parameter**		
Working volume	10 L	10 L
Temperature	25 ± 0.8 °C	25 ± 0.8 °C
pH	8.46 ± 0.5	8.46 ± 0.5
Aeration rate	2.16 ± 0.10 L/min	2.16 ± 0.10 L/min
Illumination intensity	8400 lux	8400 lux
Voltage gradient		0.62 ± 0.02 V/cm
Current density		0.261 A/m^2^
Electrodes surface area		0.015 ± 0.008 m^2^
Electrodes distance		0.03 m

**Table 3:** The removal efficiency of MPBRs with *Chlorella vulgaris*.

Source of Water/Wastewater	Type of MPBR	Influent Concentration (mg/L)	Organic Loading Rate (mg/L·d)	HRT and SRT (d)	Membrane Pore Size	Removal Efficiency%	Ref.
Synthetic municipal wastewater effluent	MPBR	TN: 25 ± 2 TP: 3.5 ± 0.4	TN: 10.58 ± 1.02 TP: 1.48 ± 0.2	HRT: 2.5 SRT: 30	0.4 µm	68 ± 3 of TN 41.81 ± 0.05 of TP	This Study
Synthetic municipal wastewater effluent	EK-MPBR	TN: 25 ± 2 TP: 3.5 ± 0.4	TN: 10.58 ± 1.02 TP: 1.48 ± 0.2	HRT: 2.5 SRT: 30	0.4 µm	43 ± 2 of TN 97.98 ± 0.02 of TP	This Study
Synthetic municipal wastewater	MPBR	N/A		HRT: 2.5 d SRT:12.5 d	N/A	50 of TN 50 of TP	[59]
Synthetic municipal wastewater	MPBR	TN: 14.1 ± 0.5 TP: 2.5 ± 0.2		HRT: 1 d SRT: 9 d	0.04 μm	31 of TN 30 of TP	[43]
Synthetic municipal wastewater	MPBR	TN: 14.1 ± 0.5 TP: 2.5 ± 0.2		HRT: 1 d SRT: 30 d	0.04 μm	32 of TN 25 of TP	[43]

## Data Availability

Data are available upon reasonable request.
